# A guideline for the management of specific situations in polycythaemia vera and secondary erythrocytosis: A British Society for Haematology Guideline

**DOI:** 10.1111/bjh.15647

**Published:** 2018-11-13

**Authors:** Mary F. F. McMullin, Adam J. Mead, Sahra Ali, Catherine Cargo, Frederick Chen, Joanne Ewing, Mamta Garg, Anna Godfrey, Steven Knapper, Donal P. McLornan, Jyoti Nangalia, Mallika Sekhar, Frances Wadelin, Claire N. Harrison, the British Society for Haematology Guideline

**Affiliations:** ^1^ Centre for Medical Education Queen's University Belfast UK; ^2^ MRC Molecular Haematology Unit MRC Weatherall Institute of Molecular Medicine Radcliffe Department of Medicine University of Oxford Oxford UK; ^3^ Castle Hill Hospital Hull and East Yorkshire Hospitals NHS Trust Hull UK; ^4^ Leeds Teaching Hospitals NHS Trust Leeds UK; ^5^ The Royal London Hospital Bart's Health NHS Trust London UK; ^6^ Birmingham Heart of England NHS Foundation Trust Birmingham UK; ^7^ University Hospital of Leicester NHS Trust Leicester (BSH representative) UK; ^8^ Department of Haematology and Haematopathology and Oncology Diagnostic Service Cambridge University Hospitals NHS Foundation Trust Cambridge UK; ^9^ Cardiff University School of Medicine Cardiff UK; ^10^ Guy's and St Thomas’ NHS Foundation Trust London UK; ^11^ Wellcome Trust Sanger Institute Cambridge UK; ^12^ Royal Free London NHS Foundation Trust University College London Hospital London UK; ^13^ Nottingham University Hospital Nottingham UK

**Keywords:** polycythaemia vera, secondary erythrocytosis, congenital erythrocytosis thrombosis, haemorrhage, pregnancy

## Methodology

This guideline was compiled according to the British Society for Haematology (BSH) process at b‐s‐h.org.uk. The Grading of Recommendation Assessment, Development and Evaluation (GRADE) nomenclature was used to evaluate levels of evidence and to assess the strength of the recommendations. The GRADE criteria can be found at https://www.gradeworkinggroup.org.

### Literature review details

The literature review was conducted on 2 March 2017. Databases searched include MEDLINE (OVID), Embase (OVID) and CENTRAL (The Cochrane library) using search terms (and relevant MESH terms) polycythaemia vera, erythrocytosis, familial, high oxygen affinity haemoglobin, defects of oxygen sensing pathway, diagnosis, investigation, molecular, mutation, *JAK2*,* MPL*,* CALR*, bone marrow, red cell mass, erythropoietin, risk, management, treatment, cytoreduction, venesection, hydroxyurea, interferon, busulfan, pipobroman, radioactive phosphorus, aspirin, anagrelide, ruxolitinib, thrombosis, haemorrhage, pregnancy, pruritus surgery and management. The search covered the period from 2005, the date of last version of the guideline (McMullin *et al*, 2005), to February week 3 2017. Exclusions included articles not in English, studies not in humans, single case reports and small case series. A total of 6062 articles were identified which, with exclusions and duplications, resulted in 1215 articles which were reviewed.

### Review of manuscript

Review of the manuscript was performed by the BSH Guidelines committee General Haematology Task Force, the BSH Guidelines Committee and the General Haematology sounding board of BSH. It was also placed on the members section of the BSH website for comment. A patient representative from MPN‐Voice (www.mpnvoice.org.uk) participated in the guideline writing meeting. The guideline has been reviewed by MPN‐Voice; this organisation does not necessarily approve or endorse the contents.

## Introduction

The previous BSH guideline for the management of erythrocytosis was published in 2005 (McMullin *et al*, 2005) and amended in 2007 (McMullin *et al*, 2007). Here, we re‐evaluate the literature formulate guidance on the management of specific situations encountered in polycythaemia vera (PV) and the management of the other types of secondary erythrocytosis. Recommendations for the diagnostic pathway of investigation of an erythrocytosis, risk stratification and management of PV are in the accompanying guideline (McMullin *et al*, 2018). We review evidence and outline guidance on management of acute thrombotic events and secondary prevention of thrombosis in PV. The unusual thrombotic events, splanchnic vein and cerebral vein thromboses are discussed and haemorrhage. The specific situations of surgery and pregnancy and guidance on management of pruritus are included. The evidence for the management of other causes of erythrocytosis, including idiopathic erythrocytosis, congenital erythrocytosis, hypoxic pulmonary disease and post‐transplant erythrocytosis, is reviewed and recommendations made.

## Management of specific situations in polycythaemia vera

### Thrombosis

Thrombotic events are the major cause of morbidity and mortality in PV, and their prevention is the main objective of treatment. About one‐third of patients present with a thrombotic event (Elliott & Tefferi, 2005; Hultcrantz *et al*, 2015; Kaifie *et al*, 2016) and younger patients diagnosed with PV have an increased risk of early death from cardiovascular disease over the general population, accounting for 45% of all deaths in PV (Marchioli *et al*, 2005; Hultcrantz *et al*, 2015).

Arterial thrombosis involving large arteries and peripheral vascular disease are more common than venous thromboembolism in PV. A prospective study (Marchioli *et al*, 2005) found that three quarters of PV patients who experience a thrombotic event had arterial and one quarter venous thrombosis.

Concerning the management of thrombosis: cardiovascular risk factors should be assessed and controlled, particularly hyperlipidaemia and hypertension, smoking and diabetes mellitus. The long‐term cardiovascular risk should be formally assessed at baseline and annually using a validated score, such as that determined by the computerised scoring tool, QRISK, although the impact of myeloproliferative neoplasms (MPN) on this score is not defined (National Institute for Health and Care Excellence [NICE], 2014). This assessment is usually conducted in primary health care, but the results should be used in primary and secondary care to optimise the management of vascular risk factors.

Risk stratification for thrombosis identifies age ≥65 years and previous thrombosis as high‐risk factors for further thrombosis. The role of thrombocytosis as a risk factor in PV is less clear than in essential thrombocythaemia (ET) (Di Nisio *et al*, 2007). The mechanism behind thrombosis is complex and factors include functional cellular changes, such as increased leucocyte‐platelet aggregation and endothelial activation, and the overexpression of activation and adhesion molecules by the neoplastic cells (Falanga & Marchetti, 2012). Some of these factors may explain the increased incidence of thrombosis at unusual sites, such as splanchnic vein thrombosis (SVT) and cerebral venous thrombosis (CVT).

Factor V Leiden (*F5* R506Q) and prothrombin gene mutation (*F2* G20210A) have been associated with thrombosis in PV and ET (Trifa *et al*, 2014). However, routine testing for inherited or acquired thrombophilia is not recommended as it has is no impact on treatment and standard guidelines for thrombophilia testing should be applied for PV patients.

Both primary and secondary prevention should include control of cardiovascular risk factors in accordance with current recommendations (NICE, 2014). Of interest, there is some evidence that angiotensin‐converting enzyme inhibitors (ACEi), as anti‐hypertensive agents, may have an additional role in controlling the haematocrit (Hct) (Barbui *et al*, 2017). In accordance with evidence for the management of PV, low‐dose aspirin should be offered to all patients (Landolfi *et al*, 2004). Low‐dose aspirin, which describes various doses (e.g. 75, 81 or 100 mg) used in different countries, is the only dose to have been assessed in recent clinical trials. The risk of major bleeding, particularly gastro‐intestinal, increases with age, and proton pump inhibitors should be given to patients over 75 years of age as well as to those a with high bleeding risk. The role of the ADP‐receptor antagonist, clopidogrel, in the long‐term prevention of MPN‐related thrombosis has not been investigated although it is widely used as an alternative in those who cannot tolerate aspirin. The Hct should be maintained below 0·45 in accordance with evidence from the Cytoreductive therapy in PV (CYTO‐PV) study (Marchioli *et al*, 2013).

### Management of acute thrombotic events

Acute thrombotic events should be managed according to current guidelines. Cytoreductive therapy should be instituted to ensure control of blood counts to the therapeutic target. Venesection to reduce the Hct to <0·45 should be undertaken if it is not controlled at the time of acute thrombosis. For arterial thrombosis, low dose aspirin should be offered to all patients, and specialist advice should be sought.

For venous thromboembolism (VTE), anticoagulation should be commenced. Low molecular weight heparin (LMWH) remains the first choice in the acute setting, followed by vitamin K antagonists (VKA) as per guidelines (NICE, 2012). Direct oral anticoagulants (DOACs) are increasingly used in the non‐MPN population for prophylaxis and VTE therapy. However, there is limited data for their specific use in MPN, where they appear to be safe and efficacious (Ianotto *et al*, 2017).

### Secondary prevention of thrombosis

Indefinite anticoagulation for VTE is recommended because of the presence of continuing risk (NICE 2012; Watson *et al*, 2015; Kearon *et al*, 2016). In MPN, the role of continuing anticoagulation, particularly in VTE, has been assessed by several studies. Around a third of patients who stop anticoagulation after initial treatment suffer a recurrence (Barosi *et al*, 2013; Kaifie *et al*, 2016). Two retrospective studies (Hernández‐Boluda *et al*, 2015; De Stefano *et al*, 2016a) showed that cessation of VKA after 3 months of treatment for a first VTE in MPN patients increased the risk of recurrent thrombosis compared with continued treatment. The later study also suggested a role for VKA in reducing the risk of arterial thrombotic recurrence and the possible benefit of adding aspirin to VKA to prevent recurrent arterial thrombosis. We are unable to make a specific recommendation about the addition of aspirin to VKA when a secondary thrombosis occurs in a patient already on VKA, as there is not yet evidence to support this. However, trials of this combination are in progress in other conditions and evidence may emerge.

For unprovoked VTE, where there is no physical precipitating factor, we therefore recommend indefinite anticoagulation to reduce the incidence of recurrence. In a significant number of patients with recurrent thrombosis (De Stefano *et al*, 2008), cell counts were sub‐optimally controlled. Cytoreduction should therefore be reviewed to ensure optimal counts are achieved. For recurrent arterial events, specialist advice should be sought from cardiovascular, stroke or vascular physicians.

### Recommendations: thrombosis

- **Patients should be screened for hypertension, hyperlipidaemia, diabetes mellitus and a smoking history. (GRADE 1B)**
- **Cardiovascular risk should be assessed at baseline and annually using a validated score such as QRISK score. (GRADE 2C)**

**Thrombotic event**

- **Treatment of acute arterial or venous thrombosis as per specialist guidelines. (GRADE 1A)**
- **Institute cytoreductive therapy to optimise count control. (GRADE 1A)**

**Secondary prevention**

- **Indefinite anticoagulation should be initiated for unprovoked venous thromboembolism dependent on bleeding risk. (GRADE 2C)**
- **Cytoreduction and venesection to keep haematocrit (Hct) <0·45. (GRADE 1A)**

### Splanchnic vein thrombosis

Unusual sites of venous thrombosis are more common in patients with MPN, especially in younger patients. The splanchnic circulation is particularly vulnerable in patients with *JAK2* V617F mutation. SVT comprises thrombosis of hepatic, portal, superior mesenteric and splenic veins singly or in combination. The incidence of SVT during the course of PV ranges from 0·8 to 7·8% (Sekhar *et al*, 2013). Overall, portal‐mesenteric axis venous thrombosis is commoner than hepatic vein thrombosis or Budd Chiari Syndrome (BCS), however BCS is more prevalent in PV (Smalberg *et al*, 2012). The mortality of MPN‐SVT is high, at 20–40% at 10 years, due to the index occurrence or recurrent thrombosis, bleeding or leukaemic transformation (Hoekstra *et al*, 2011; Ageno *et al*, 2015).

The presence of hypersplenism, SVT‐related haemodilution and iron deficiency due to blood loss and/or masked polycythaemia can artificially lower the blood counts and a normal Hct may be found despite a high red cell mass (Kiladjian *et al*, 2008). Therefore, it is important to investigate all patients presenting with SVT without attributable local pathology, such as malignancy, sepsis or pancreatitis, for the presence of *JAK2* V617F mutation, which is present in 80–90% of patients with MPN‐SVT (Kiladjian *et al*, 2008), and if this is negative, testing should be done for a *CALR* mutation, which is present in about 2·5% (Sekhar *et al*, 2016).

Although PV is commoner in males, PV‐related SVT is commoner in young females. The reason for the predilection of splanchnic venous circulation to thrombosis in MPN is not clear. Many series report co‐existing inherited or acquired thrombophilia in 25–30% patients, especially protein S deficiency which has been associated with unusual site thrombosis in MPN patients (Gisslinger *et al*, 2005). In patients with SVT or cerebral vein thrombosis (CVT), testing for inherited and acquired thrombophilia should be considered if it would influence management decisions, such as intensity and duration of anticoagulation in the face of higher risks of bleeding (Tait *et al*, 2012).

Anticoagulation treatment in the acute phase should be undertaken with LMWH unless there are compelling contra‐indications (Tait *et al*, 2012). In addition, the treatment of acute phase SVT includes interventions to recanalize the blocked veins, such as thrombolysis and insertion of stents. Optimal management results in long‐term survival of 85% at 10 years in BCS. Patients requiring liver transplantation have a high mortality (Potthoff *et al*, 2015). The management of PV‐related SVT therefore needs close coordination between hepatologists, interventional radiologists and haematologists to achieve an accurate diagnosis, anti‐coagulate effectively, undertake interventional procedures and optimise cytoreduction in the acute phase.

Recurrence risk is high and prevention is important because of significant morbidity and mortality associated with recurrence. Overall 25–30% of MPN‐SVT patients suffer a recurrence by 10 years (Hoekstra *et al*, 2011; Ageno *et al*, 2015). Although recurrence is more likely in the splanchnic circulation (Amitrano *et al*, 2007), it can occur in other venous or arterial locations.

Anticoagulant practice in MPN‐SVT is heterogeneous (Ellis *et al*, 2014). Current VTE guidelines recommend continuing anticoagulation in the presence of continuing active risk (Kearon *et al*, 2012; Tait *et al*, 2012). On this basis, PV‐related SVT is an indication for long‐term anticoagulation. Several prospective and retrospective registry studies conclude that long‐term anticoagulation reduces recurrence risk at splanchnic and other sites with an acceptable level of bleeding complications (Amitrano *et al*, 2007; Ageno *et al*, 2015; De Stefano *et al*, 2016b). Recurrence in the arterial circulation has been observed in patients not on anti‐platelet agents (Hoekstra *et al*, 2011; De Stefano *et al*, 2016b). The strategy of using anti‐platelet agents in addition to VKA is followed in selected patients with BCS with stents *in‐situ* but registry data do not suggest any benefit from this combination for prevention of recurrence of thrombosis outside of this group. Concerning anticoagulation with DOACs, 20% of patients with non‐cirrhotic SVT suffered thrombosis or haemorrhage at 2 years (De Gottardi *et al*, 2017). These agents have not been studied in MPN‐related SVT where LMWH followed by warfarin is the standard of care.

The impact of cytoreduction in MPN with SVT has not been systematically studied. Cytoreduction was used in 40–70% patients across registries and did not uniformly influence recurrence risk. However, abnormally high blood counts due to inadequate cytoreduction were present in over half the patients with recurrent thrombosis (De Stefano *et al*, 2016b) indicating the need for rigorous clinical monitoring of patients on cytoreduction. Venesection alone is not appropriate to treat PV and cytoreduction should be undertaken in the presence of SVT. Patients with PV‐related SVT are younger and interferon may be suitable as a first line agent, however data on interferon in this group of patients are not yet available. There are no data to guide therapeutic targets of cytoreductive treatment. In particular, whether more aggressive cytoreduction offsets the residual risk of recurrent thrombosis is not known. One strategy is to use standard targets for treatment of PV with an aim to achieving normal Hct, white blood cell (WBC), neutrophil and platelet counts (Barbui *et al*, 2011). An alternative strategy is to use lower targets, as defined by experts in the ongoing trial of pegylated interferon 2 alpha in MPN including MPN‐SVT aiming for a Hct ≤0·42, WBC count between 2 and 8 × 10^9^/l, and platelet count between 100 and 200 × 10^9^/l (Hoffman, 2012). As this study is not yet complete the evidence for the use of these targets is weak. Nevertheless, the fact that these patients do develop thrombosis with normal counts suggests functional thrombogenicity, which may be altered by using disease‐modifying agents to achieve these lower targets. Small doses of cytoreductive agents are often sufficient to achieve therapeutic targets and care must be taken to avoid significant thrombocytopenia in the face of continuing anticoagulation. Treatment with ruxolitinib was found to reduce the volume of spleen in a third of MPN‐SVT patients after 2 years (Pieri *et al*, 2017).

Complications of treatment include bleeding, especially gastrointestinal haemorrhage and, in particular, variceal haemorrhage (Ageno *et al*, 2015; De Stefano *et al*, 2016b). A quarter of major bleeds are intracranial. Other than thrombocytopenia, cytoreduction does not have any impact on bleeding risk. Bleeding risk can be minimized by careful management of oesophageal varices and concurrent use of proton pump inhibitors.

### Cerebral vein thrombosis

Cerebral vein thrombosis occurs in about 1% of MPN patients, primarily in those with ET bearing the *JAK2* V617F mutation (Martinelli *et al*, 2014). In selected patients with unexplained CVT, testing for *JAK2* V617F can identify the aetiology and assist management. Recurrence rates are higher in MPN‐CVT, especially in spontaneous CVT, and a third of patients suffer recurrence despite anticoagulation and cytoreduction (Miranda *et al*, 2010; Martinelli *et al*, 2014). Recurrence is systemic, affecting venous and arterial circulation and often involves splanchnic veins. Patients with PV‐related CVT should be treated with long‐term anticoagulation and cytoreduction. The role of aspirin and JAK inhibitors have not been studied in this group of patients.

### Recommendations: splanchnic vein thrombosis and cerebral vein thrombosis

- **Patients presenting with splanchnic vein thrombosis (SVT) not associated with local malignancy should be tested for the presence of** ***JAK2*** **V617F mutation and if negative,** ***CALR*** **mutation. (GRADE 1A)**
- **Patients with SVT should be treated with long‐term anticoagulation. (GRADE 1B)**
- **Patients with CVT should be treated with long‐term anticoagulation. (GRADE 2C)**
- **Cytoreduction and control of blood counts should be undertaken in keeping with management of high‐risk polycythaemia vera. (GRADE 1A)**
- **Patients should be managed in a multidisciplinary setting in conjunction with interventional radiology and hepatology. (GRADE 1B)**

## Haemorrhage

Haemorrhage is both a less frequent and generally less severe clinical complication of PV than thrombosis. The reported incidence varies greatly between studies (Elliott & Tefferi, 2005; Marchioli *et al*, 2005; Kander *et al*, 2015; Kaifie *et al*, 2016). The principal sites affected are skin, mucous membranes and gastrointestinal tract. Patients with SVT are at a particularly high risk of bleeding from gastro‐oesophageal varices.

Factors contributing to bleeding in patients with PV include extreme thrombocytosis (platelet counts >1500 × 10^9^/l) (Finazzi *et al*, 1996) and associated acquired von Willebrand syndrome (aVWS). This has been reported to affect 12% of PV patients, usually those with high platelet counts, and does not always correlate with bleeding (Kander *et al*, 2015; Mital *et al*, 2015). Other platelet function defects are reported in PV but they are not predictive of bleeding. However, the risk of bleeding is likely to be higher with combined use of anti‐platelet therapies, such as dual anti‐platelet therapy or anti‐platelet therapy and VKA (Hallas *et al*, 2006; Kaifie *et al*, 2016). Other studies have reported splenomegaly (Kaifie *et al*, 2016) and a high leucocyte count (Chou *et al*, 2013; Lim *et al*, 2015) as predictors of haemorrhage.

Blood counts should therefore be optimised. Other measures to consider include adjustment of any concomitant anti‐platelet and/or anticoagulant therapy. Clinically significant bleeding may, paradoxicall,y require platelet transfusion (Terasako & Sasai, 1998) and a role for tranexamic acid has been suggested (Spivak, 2002). The utility of recombinant activated factor VII (rFVIIa) is unknown in MPN patients with uncontrolled life‐threatening bleeding and perhaps worthy of further study.

### Recommendations: haemorrhage

- **Screen for acquired von Willebrand syndrome (aVWS) if a bleeding history is present. If negative, then test for platelet function defect and consult a haemostasis expert. (GRADE 2C)**
- **Be cautious with the use of anti‐platelet drugs/anticoagulants in patients with extreme thrombocytosis but balance the thrombotic and bleeding history of the patient. (GRADE 2C)**
- **Manage significant bleeding episodes with tranexamic acid and/or platelet transfusion; cease/reduce aspirin. (GRADE 2C)**
- **Optimise cytoreductive treatment. (GRADE 2B)**

### Peri‐operative management

Polycythaemia vera patients undergoing surgery are, paradoxically, at risk of both haemorrhage and thrombosis. Even in treated PV patients with well controlled counts, there is an increased risk of haemorrhage, demonstrated by their increased transfusion requirements (Weingarten *et al*, 2015) compared to non‐PV patients (Ruggeri *et al*, 2008). VTE incidence was increased 5‐fold despite prophylaxis. It is therefore important that PV patients are assessed pre‐operatively and abnormal counts optimised, balancing the acute need for surgical procedure against the risk of bleeding and thrombosis.

Polycythaemia vera patients who bleed may have significant qualitative platelet abnormalities. Patients with a prior history of bleeding should be tested for platelet function and for aVWS preoperatively. Anti‐thrombotic prophylaxis should be administered according to standard postoperative guidelines. There is a little evidence that use of cytotoxic agents impairs wound healing: their use in the 4–6 weeks after surgery needs to be considered based on thrombotic and haemorrhagic risk and could be avoided if venesection alone is sufficient.

### Recommendations: surgery

- **Pre‐operative planning should involve a haematologist to optimise count control and individualise peri‐operative plan. (GRADE 1B)**
- **Use standard protocols for managing antithrombotic prophylaxis. (GRADE 1B)**
- **If the patient has a bleeding history, screen for coagulation tests, aVWS and platelet function tests pre‐operatively. (GRADE 2C)**

## Pregnancy

Polycythaemia vera is uncommon in females of reproductive age, occurring in less than 0·3 per 100 000 (Srour *et al*, 2016). There are only small case series describing the management of pregnancy in patients with PV (Harrison, 2005; Robinson *et al*, 2005; Griesshammer *et al*, 2008). Larger case series are published for pregnancy management in patients with ET or cohorts of patients with all MPNs (Alimam *et al*, 2016; Skeith *et al*, 2017). The guidance detailed here refers to these case series, practices extrapolated from the management of pregnant patients with ET and personal practice.

Pregnancy is a prothrombotic state with increased risk of thromboembolism in patients with PV. Consequently, there is a significant risk of obstetric complications, such as fetal loss throughout all trimesters, intra‐uterine growth retardation, prematurity, maternal thromboembolism and haemorrhage. Previously significant fetal loss and maternal morbidity was seen, but a recent prospective study of pregnancy outcomes in MPNs showed better outcomes. There were no maternal deaths or thrombotic events (Alimam *et al*, 2016). This improvement in pregnancy outcomes is probably partly due to a more protocol‐based management and a multidisciplinary approach.

Pregnancies in patients with PV should be managed under the joint care of an obstetrician experienced in the care of high‐risk patients and a haematologist experienced in MPNs. Management should start with preconceptual planning for both male and female patients. The Hct should be kept within a gestational‐appropriate range and potentially teratogenic medications (such as hydroxycarbamide) should be stopped a minimum of 3 months prior to planned conception in males and females. There is insufficient data regarding the safety of anagrelide during pregnancy and it should therefore be withdrawn at least 3 months prior to conception in females. Interferon should be considered for those patients who require cytoreductive therapy, though patients should be counselled regarding the potential for interferon to reduce fertility. All patients should receive low dose aspirin (unless there are patient‐specific contraindications) throughout pregnancy and the postpartum period.

Patients with high risk pregnancies should be identified (Table 1). These are patients with previous arterial or venous thrombosis or haemorrhage attributed to PV, previous pregnancy complications (>3 first trimester losses, >1 s or third trimester loss, birth weight <5th centile for gestation, intrauterine death or stillbirth, pre‐eclampsia), extreme thrombocytosis before or during pregnancy, diabetes mellitus or hypertension requiring pharmacological treatment.

**Table 1:** Management of risk factors during pregnancy

	During pregnancy	6 weeks postpartum
High risk pregnancy		
Previous history of arterial thrombosis due to PV	Commence IFN Prophylactic dose LMWH twice daily (e.g. enoxaparin 40 mg bd) Aspirin	Reduce LMWH to once daily prophylactic dose (e.g. enoxaparin 40 mg od) and Aspirin Decision to continue IFN based on individual patient discussion
Previous history of venous thrombosis due to PV, previous pregnancy complications (>3 first trimester losses, >1 s or third trimester loss, birth weight <5th centile for gestation, intrauterine death or stillbirth, pre‐eclampsia)	Commence IFN Once daily prophylactic dose LMWH (e.g. enoxaparin 40 mg od) Aspirin from confirmation of pregnancy LMWH increasing to twice daily from 16 to 20 weeks’ gestation	Continue once daily prophylactic dose LMWH (e.g. enoxaparin 40 mg od) and Aspirin Decision to continue IFN based on individual patient discussion
Previous history of haemorrhage due to PV or significant antepartum or postpartum haemorrhage requiring transfusion	Commence IFN Addition or continuation of aspirin to be decided on a patient‐specific basis	Continuation of aspirin and Addition of once daily prophylactic dose LMWH on a patient‐specific basis Decision to continue IFN based on individual patient discussion
Thrombocytosis, platelet count >1500 × 10^9^/l before or during pregnancy and/or diabetes mellitus or hypertension requiring pharmacological treatment	Commence IFN Continue aspirin	Continue once daily prophylactic dose LMWH (e.g. enoxaparin 40 mg od) and Aspirin Decision to continue IFN based on individual patient discussion
Standard risk pregnancy		
All other pregnancies	Aspirin	Once daily prophylactic dose LMWH (e.g. enoxaparin 40 mg od) and Aspirin

IFN, interferon; LMWH, low molecular weight heparin; PV, polycythaemia vera.

Those patients with a prior history of thrombosis and/or fetal morbidity should commence cytoreduction therapy with interferon and prophylactic LMWH in addition to aspirin. The dose and frequency of LMWH should be adjusted according to renal function, body weight and previous history. For those patients of normal body weight, no renal impairment and with a history of previous venous thrombosis or fetal morbidity, standard dose thromboprophylaxis (e.g. enoxaparin 40 mg/day) should be commenced as soon as pregnancy is confirmed. This should be increased to intermediate dose thromboprophylaxis (e.g. twice daily enoxaparin 40 mg) at 16–20 weeks. Patients with a previous history of arterial thrombosis should be commenced on intermediate dose prophylaxis (e.g. twice daily enoxaparin 40 mg) throughout pregnancy (Harrison, 2005). Interferon should be initiated for those patients with a history of PV‐associated haemorrhage, extreme thrombocytosis (>1500 × 10^9^/l) before or during pregnancy and/or diabetes mellitus or hypertension requiring pharmacological treatment. All other patients (standard risk pregnancy) should receive low dose aspirin throughout pregnancy with the addition of once daily prophylactic dose LMWH for 6 weeks postpartum.

During pregnancy, the Hct should be maintained within the normal range for gestation with venesection (Table 2). Interferon can be considered if venesection fails to adequately control the Hct or is not tolerated. Patients should be reminded to avoid iron supplementation in the absence of proven iron depletion. Where supplementation is offered, this should be at a low dose with regular monitoring. The full blood count, blood pressure and urinalysis should be checked every 4 weeks until 24 weeks and then every 2 weeks. Fetal growth should be assessed by regular ultrasound. Uterine Dopplers should be performed at 20 weeks’ gestation to assess placental function by measuring the mean pulsatile index. If the index is >1·4, the frequency of growth scans should be increased and consideration given to escalating treatment to include LMWH and interferon.

**Table 2:** 95% range for haematocrit during pregnancy (taken from Bain, 2015)

	1st trimester	2nd trimester	3rd trimester
Haematocrit	0·31–0·41	0·30–0·38	0·28–0·39

Dehydration should be avoided in all patients during labour and the third stage of labour should be actively managed. Thromboembolic deterrent (TED) stockings are advisable during labour and if immobile during the postpartum period. LMWH and aspirin should be stopped prior to elective caesarean section or once spontaneous labour has begun, as per local guidelines.

Low molecular weight heparin should be restarted at the earliest opportunity post‐partum provided there is no significant bleeding. The dose of LMWH should be once daily and should be continued for 6 weeks post‐partum. Aspirin should be continued throughout the post‐partum period. Breastfeeding is safe with both aspirin and LMWH, and permissible with interferon. The Hct should be monitored in the post‐partum period and maintained at less than 0·45. Attention should be paid to the platelet count, as a rebound thrombocytosis may develop, necessitating the introduction of cytoreductive therapy.

There is no evidence regarding the management of PV during fertility treatment. Ovarian hyperstimulation is associated with an increased risk of thrombosis and therefore cytoreductive therapy (with interferon) and thromboprophylaxis with LMWH should be considered.

### Recommendations: pregnancy management

- **There should be close collaborative management between obstetrician and haematologist to formulate an individualised plan for the pregnancy, delivery and postpartum period based on the previous history of thrombosis, haemorrhage and previous pregnancies. (GRADE 1C)**
- **Avoid cytoreductive therapy (such as hydroxycarbamide and anagrelide) for a minimum of 3 months in male and female patients prior to planned conception and in the first trimester. Commence interferon if cytoreduction is required. (GRADE 1C)**
- **Maintain Hct within the normal range for gestation. (Grade 1C)**
- **Consider low molecular weight heparin (LMWH) and/or interferon in addition to aspirin for high risk pregnancies (and for 6 weeks postpartum), based on individual patient discussion. (GRADE 1C)**
- **Commence prophylactic dose LMWH for 6 weeks postpartum for standard risk pregnancies. (GRADE 1C)**
- **Assess growth scans regularly and mean pulsatile index with uterine Doppler scans at 20 weeks. Consider escalation of treatment to include LMWH and interferon if scans are abnormal. (GRADE 1C)**
- **The risk of continuing interferon whilst breastfeeding is permissible and should be individually considered. There are no contraindications to breastfeeding whilst taking aspirin and LMWH. (GRADE 1C)**

## Pruritus

Pruritus is common in PV, occurring in up to 85% of patients (Mesa *et al*, 2017). Pruritus can predate or accompany the diagnosis of PV (Le Gall‐Ianotto *et al*, 2017). It can occur spontaneously or be precipitated by water or changes in temperature and can have a significant negative impact on quality of life, affecting sleep, participation in social activities and bathing (Siegel *et al*, 2013). The intensity of pruritus varies but can be severe causing emotional depression, anxiety and even suicide ideation.

The pathogenesis of pruritus in PV is not clear. Mechanisms involving basophils (Pieri *et al*, 2009) and mast cells (Ishii *et al*, 2009) have been postulated. It can also be associated with the iron deficiency that commonly results from frequent venesection therapy. Pruritus has been found to be correlated with granulocyte *JAK2* V617F homozygosity and allele burden (Vannucchi *et al*, 2007; Gangat *et al*, 2008). The mechanism for this association is unclear though it may relate to cytokine levels.

The management of pruritus is challenging. Symptoms can improve or resolve with control of blood counts, using venesection alone or with cytoreductive therapy (Le Gall‐Ianotto et al, 2017). Improvement in pruritus has been reported with hydroxycarbamide (Sharon *et al*, 1986), interferon (although this drug can also worsen pruritus) (Lengfelder *et al*, 2000), busulfan and ^32^P (Jackson *et al*, 1987). Trials of ruxolitinib in PV have shown this agent to be effective in controlling pruritus in patients who have failed to respond to hydroxycarbamide (Vannucchi, 2015; Mesa *et al*, 2017; Passamonti *et al*, 2017). In those who are severely iron deficient as a result of venesection, pruritus may improve with iron replacement, if this is possible, but iron replacement must be undertaken with extreme caution and close supervision of blood counts.

However, pruritus can persist in patients with PV once their blood counts have normalised (Gangat *et al*, 2008). Antihistamines (both H1 and H2 receptor antagonists) are commonly prescribed though demonstrate mixed results (Weick *et al*, 1982; Steinman & Greaves, 1985) and combination therapy at high doses may be required to be effective. Other adjunctive therapy that have been shown to be of benefit include selective serotonin reuptake inhibitors (SSRIs) (Tefferi & Fonseca, 2002) and anticonvulsants (such as gabapentin and pregabalin). Tricyclic antidepressants (Weisshaar *et al*, 2012), thalidomide and naltrexone have also been found to be beneficial (Phan *et al*, 2010). Sometimes creams containing menthol can also provide some relief (Patel *et al*, 2007). From a practical point of view, it is advisable to cut nails to reduce skin damage. Joint management with a dermatologist should be considered for refractory symptoms and for those patients requiring narrow‐band‐ultraviolet (UVB) or ultraviolet A (UVA) (Rivard & Lim, 2005). The addition of oral psoralen to UV light (PUVA) is only occasionally used. In patients who do not require cytoreductive therapy, the above options should be tried prior to initiating cytoreductive therapy just to control pruritus.

### Recommendations: pruritus

- **Use antihistamines which may require high doses and multiple agents. (GRADE 1C)**
- **Consider addition of H2 antagonists (such as ranitidine or cimetidine) for dual‐histamine blockade. (GRADE 2C)**
- **Consider additional adjunctive therapies such as tricyclic antidepressants, selective serotonin reuptake inhibitors and anticonvulsants (gabapentin, pregabalin) for resistant pruritus. (GRADE 2C)**
- **Consider cytoreductive therapy if pruritus fails to respond to control of Hct with venesection alone or alternative therapies. (GRADE 1C)**
- **Ruxolitinib can be effective in controlling pruritus that fails to respond to hydroxycarbamide or interferon. (GRADE 1B)**
- **Consider referral to a dermatologist for consideration of PUVA if pruritus is refractory to pharmacological agents. (GRADE 2C)**

## Management of secondary erythrocytosis

### Idiopathic erythrocytosis

Idiopathic erythrocytosis (IE) is a diagnosis of exclusion. It is an absolute erythrocytosis of no identifiable cause that is more frequent in males (Randi *et al*, 2016). Every effort should be made to exclude identifiable primary and secondary causes of erythrocytosis (including PV and congenital erythrocytosis) before a diagnosis of IE is made. Erythropoietin (EPO) levels are unhelpful as they are below normal in a third of patients, suggesting a primary erythrocytosis, and elevated in two‐thirds, suggesting a secondary erythrocytosis. Recent studies using sequencing analysis have identified a number of patients who were previously diagnosed with IE as having mutations in either erythrocytosis‐associated genes or novel genes (Bento *et al*, 2014; Camps *et al*, 2016), suggesting that at least some of these cases have a genetic basis.

Although previous data suggested that the risk of thrombosis and/or haemorrhage was considerably elevated in IE, these observations were based on old evidence that predated the identification of the *JAK2* V617F mutation and therefore some patients with PV are likely to have been included in the study cohorts. More contemporary data suggests that the risk of thrombosis/bleeding is low in IE (Bertozzi *et al*, 2017). There are no clinical studies evaluating the use of aspirin or venesection in IE and therefore evidence‐based recommendations cannot be made. Cytoreductive therapy is not indicated in patients with IE. The thrombotic risk factors should be evaluated in each case and, in selected cases, the Hct can be controlled by venesection. There is no evidence on which to determine the target Hct as relevance of data from PV patients for IE is unclear. A pragmatic approach is required for these patients with rigorous control of vascular risk factors, such as diabetes mellitus, hypertension and smoking and use of aspirin in cases where this would be otherwise clinically indicated for primary or secondary prevention. It would be reasonable to venesect patients with an arbitrary target Hct of <0·55, although a lower target Hct of <0·45 may be appropriate for a patient with a history of thrombosis considered to be related to the erythrocytosis.

### Recommendations: idiopathic erythrocytosis

- **Exclude primary and secondary causes of erythrocytosis. (GRADE 1B)**
- **Confirm absolute erythrocytosis. (GRADE 1B)**
- **Consider more extensive genetic testing if available. (GRADE 1B)**
- **Aspirin if otherwise clinically indicated for primary or secondary prevention. (GRADE 1B)**
- **Cytoreductive therapy is not recommended. (GRADE 1B)**
- **Control Hct with venesection in selected cases. Tailor the target Hct based on the thrombotic history and risk factors. (GRADE 2C)**

### Congenital erythrocytosis

Germ‐line defects can result in a congenital erythrocytosis, which is usually diagnosed in children or young adults, often in those with a family history of erythrocytosis. A wide range of defects have been described leading to primary and secondary erythrocytosis including high oxygen affinity haemoglobins (Bento *et al*, 2014; Camps *et al*, 2016).

These conditions are rare and clinical events and outcomes are poorly described. In Chuvash polycythaemia, where affected individuals have a homozygous mutation in the *VHL* gene increased thrombotic complications have been reported (Sergueeva *et al*, 2017). Major thrombotic events have also been reported to occur in young individuals with other types of congenital erythrocytosis (Bento *et al*, 2014). There are also reports of increased pulmonary artery pressure in both Chuvash polycythaemia and other congenital erythrocytosis (Bushuev *et al*, 2006; Smith *et al*, 2006). Recently, somatic gain‐of‐function mutations in *EPAS1* (*HIF2A*) have been associated with neuroendocrine tumours in the presence of erythrocytosis (Zhuang *et al*, 2012) and, therefore, it may be advised to screen for these events in individuals with known mutations.

There is little hard evidence to advise on management of congenital erythrocytosis. Low‐dose aspirin is of benefit in myeloproliferative neoplasms in the prevention of thromboembolic events (Landolfi *et al*, 2004) and, by extrapolation, may be of benefit in congenital erythrocytosis. Venesection can be used to reduce the Hct but it must be considered that the raised Hct is in keeping with the physiological consequences of the mutation and in Chuvash polycythaemia there are suggestions that venesection may be detrimental. However, in those with symptoms for which the raised Hct may be contributory or if a previous thrombotic episode has occurred, or in individuals who are asymptomatic but who have affected family members with the same genetic lesion and a thrombotic episode, venesection to a target of 0·52 is suggested. This advice particularly pertains to those with high oxygen affinity haemoglobins (Mangin, 2017).

In Chuvash polycythaemia the VHL protein is a SOCS1‐coperative regulator of JAK2 and JAK2‐targeted therapy may be of benefit in this disorder (Russell *et al*, 2011). Recently there have been reports of clinical improvement in patients with Chuvash polycythaemia on ruxolitinib (Zhou *et al*, 2016) and ruxolitinib may therefore be considered for the management of this specific cause of congenital erythrocytosis.

### Recommendations: congenital erythrocytosis

- **Consider low‐dose aspirin. (GRADE 2C)**
- **Consider venesection, particularly if symptoms for which raised Hct might be contributory or if previous thrombotic episode or in an asymptomatic individual in whom a family member with the same genetic lesion has had thrombotic episode (particularly with high oxygen affinity haemoglobins). (GRADE 2C)**
- **An Hct target of 0·52 is suggested (do not attempt to reduce Hct to the normal range). (GRADE 2C)**
- **Consider screening for pulmonary hypertension and neuroendoocrine tumours in those with specific mutations e.g.** ***EPAS1, VHL*** **. (GRADE 2C)**
- **Ruxolitinib should be considered in Chuvash polycythaemia. (GRADE 2C)**

### Hypoxic pulmonary disease

Erythrocytosis can be associated with advanced chronic obstructive pulmonary disease (COPD) and with obstructive sleep apnoea syndrome (OSA). In both conditions, erythrocytosis is relatively rare. In COPD, the incidence of erythrocytosis, usually defined as Hct > 0·55, ranges from 6 to 8% (Chambellan *et al*, 2005; Cote *et al*, 2007). Incidences of 1·7–8% are reported in OSA (Gangaraju, 2016; Nguyen & Holty, 2017).

The prognostic significance of erythrocytosis in hypoxic pulmonary disease (HPD) is not known but the development of an erythrocytosis in patients with HPD is associated with an increased risk of the development of cor pulmonale and poor median survival of 2–3 years (Criner, 2000). However, in a retrospective analysis of severe COPD patients on long term oxygen treatment, a Hct ≥ 0·55 was associated with better prognosis compared to COPD patients with anaemia (Chambellan *et al*, 2005; Cote *et al*, 2007).

The risk of PE in this setting is also unclear (Nadeem *et al*, 2013) as an increased Hct in the general population is also associated with increased VTE risk (Braekkan *et al*, 2010). A recent study suggests that patients with COPD at low risk of VTE had increased incidence of pulmonary embolism if they had concurrent erythrocytosis (Guo *et al*, 2016).

Additional risk factors affecting circulatory compromise and tissue oxygen delivery include carbon monoxide in smokers, extent of hypercapnia, renal blood flow, acid‐base balance (pH), capacity of the bone marrow to respond to erythropoietic drive, position on the oxygen dissociation curve and changes in the peripheral vascular circulation. Furthermore, for an individual patient, co‐existent age‐related vascular disease may also affect thrombotic risk.

The evidence base for recommendations for the management of erythrocytosis in HPD remains limited. However, treatment of the underlying hypoxia reduces the Hct. Long term oxygen therapy improves survival in patients with COPD and severe hypoxaemia (PaO_2_ below 7·3 kPa or <8·0 kPa with nocturnal hypoventilation) (Crockett *et al*, 2001).

All patients with erythrocytosis consequent upon HPD should therefore be evaluated by a respiratory physician for consideration of long‐term oxygen therapy or alternative methods of improving oxygenation. If they are smokers they should be strongly advised to stop. In addition to supplemental oxygen, nocturnal oxygenation may also be improved by the use of non‐invasive ventilation. Therefore, failure to achieve adequate oxygenation in HPD should not be accepted without review by a specialist respiratory physician. Of benefit regarding limited venesection in patients with HPD, reducing the Hct to 0·50–0·52 led to an improvement in exercise tolerance but a further staged reduction of Hct to 0·45 did not give additional benefit as discussed in the previous guideline (McMullin *et al*, 2005). However, a Hct lower than 0·45 is detrimental to these patients, and associated with poorer outcome (Chambellan *et al*, 2005). It is of interest that ACEi (Leshem‐Rubinow *et al*, 2012) have the ability to reduce the Hct in this setting.

In the case of obstructive sleep apnoea, erythrocytosis is associated with nocturnal oxygen desaturation and such patients should be referred for appropriate investigation. Long term non‐invasive continuous positive airway pressure (CPAP) has been shown to reduce erythrocytosis (Krieger, 1992).

### Recommendations: hypoxic pulmonary disease

- **Patients with hypoxic pulmonary disease who develop erythrocytosis should be evaluated by a respiratory physician for consideration of long‐term oxygen therapy or alternative therapy. (GRADE 1A)**
- **Patients who are symptomatic as a result of hyperviscosity or have a Hct >0·56 should be considered for venesection to reduce this to 0·50–0·52. (GRADE 2C)**

### Post‐transplant erythrocytosis

The definition of post‐transplant erythrocytosis (PTE) varies across publications, which impacts on the estimate of incidence. A Hct over 0·51 lasting more than a month after renal transplantation is a commonly used definition (Vlahakos *et al*, 2003); however, gender‐specific cut‐offs (males, Hct > 0·52; females >0. 50), use of haemoglobin concentration in the definition (>170 g/l) and variable duration (>1 month, 3 months, 6 months) have also been used. The incidence of post‐renal transplant erythrocytosis is between 5% and 20% and is decreasing (Kiberd, 2009). PTE also occurs in approximately 1% of patients after haematopoetic stem cell transplantation (HSCT) (Ahmed *et al*, 2011; Atilla *et al*, 2016) and after combined pancreatic–renal transplant, with a reported incidence of 16% (Guerra *et al*, 2010).

Post‐transplant erythrocytosis following renal transplantation is a benign, often self‐limiting condition which typically develops within the first year following transplantation, although it can occur at 2–4 years (Charfeddine *et al*, 2008; Kiberd, 2009). Spontaneous resolution can occur over a period of 1–4 years (Einollahi *et al*, 2005) and treatment with ACEi or angiotensin receptor blockers (ARB) successfully reduces the Hct in the majority (Charfeddine *et al*, 2008). Indeed, the increasing use of these drugs for hypertension at earlier stages after transplantation has been deemed responsible for the falling incidence of PTE (Kiberd, 2009). Risk factors for developing erythrocytosis include male gender, retained native kidney, hypertension, renal artery stenosis, a short period of pre‐transplant dialysis, reduced need for pre‐transplant use of EPO or being transfused. Studies also list good allograft function, diabetes, rejection‐free course and use of ciclosporin adversely affecting the incidence and use of mycophenolate mofetil favourably affecting the incidence (Kiberd, 2009). Symptoms due to high viscosity, comprising dizziness, malaise, headache, lethargy and plethora, are reported. No thrombosis or mortality has been reported in recent studies (Kiberd, 2009).

Venesection has been used by some in patients with persistently high Hct (>0·57–0·60) and helps reduce the Hct but does not reduce the incidence of thrombosis (Charfeddine *et al*, 2008). Aspirin use has been variable and its effect is not clear.

Mechanisms of post‐renal transplant erythrocytosis have been reviewed (Vlahakos *et al*, 2003). Recipient‐related, rather than donor‐related, features underlie the development of PTE. The erythrocytosis is EPO‐responsive, with high EPO levels at onset reducing to lower levels after suppression of the renin‐angiotensin‐ system (RAS) using ACEi, ARBs or native kidney nephrectomy; recurrence after discontinuation of these drugs is accompanied by increased EPO levels (Vlahakos *et al*, 2003). Retention of native kidneys is thought to lead to a ‘hyper‐erythropoietinaemia’. Unlike suppression of the RAS, venesection increases EPO levels. The renin‐angiotensin, androgen and adrenergic systems increase erythropoiesis and sensitivity of erythroid progenitors to EPO. The likely common pathway is via insulin‐like growth factor‐1 (IGF1) which increases erythropoiesis. ACEi reduce IGF1 levels thus reducing erythropoiesis. Pancreatic plus renal transplant increases IGF1 levels, and use of systemic venous drainage (as opposed to portal venous drainage) was associated with a higher incidence and earlier onset of PTE (Guerra *et al*, 2010). These patients also required more frequent venesection to maintain a stable Hct. Treatment with ACEi or ARB reduces Hct to a nadir level at 3 months with stable results for several years. Discontinuation usually leads to recurrence of post‐transplant erythrocytosis, but 20–30% of patients maintain a normal Hct after cessation. The use of aggressive venesection leads to iron deficiency.

Haematopoetic stem cell transplantation‐related erythrocytosis has been observed in about 1% of patients transplanted for aplastic anaemia but also in other conditions, including myelodysplastic syndromes and acute leukaemia (Ahmed *et al*, 2011; Atilla *et al*, 2016). Patients who developed PTE did not have total body irradiation and were mostly men. Erythrocytosis was asymptomatic, with no thrombosis or mortality reported. All were treated with venesection on an ongoing basis. PTE after HSCT starts, on average, about 11 months after the transplant and can be self‐limiting, lasting approximately 6 months.

Persistent post‐transplant erythrocytosis unresponsive to ACEi, ARB or venesection requires further investigation to define underlying causes, including *JAK2* V617F mutation analysis.

### Recommendations: post‐transplant erythrocytosis

- **Treat hypertension and rising Hct promptly if persistent (>1 month) and otherwise unexplained. (GRADE 2B)**
- **Use angiotensin‐converting enzyme inhibitors (e.g. captopril, enalapril) or angiotensin receptor blockers (e.g. losartan). (GRADE 2B)**
- **No benefit of aspirin. (GRADE 2C)**
- **No evidence of benefit of venesection in renal transplant but consider for persistent symptoms, target Hct 0·50. (GRADE 2C)**
- **Consider venesection in post‐ haematopoetic stem cell transplantation, aim for a Hct <0·50. (GRADE 2C)**

### Cyanotic congenital heart disease

Patients with congenital cyanotic heart disease are best managed by specialist cardiology clinics. Decisions about venesection for these patients should not be made in a haematology clinic.

## Conclusion

Using the available evidence and best practice we have suggested practical guidance for the management of specific situations and complications of polycythaemia vera. Management of the various types secondary erythrocytosis from the available evidence is outlined.

## Declaration of Interests

The BSH paid the expenses incurred during the writing of this guidance. All authors have made a declaration of interests to the BSH and Task Force Chairs which may be reviewed on request.
